# Ecological∼Enactivism Through the Lens of Japanese Philosophy

**DOI:** 10.3389/fpsyg.2020.01347

**Published:** 2020-07-29

**Authors:** Jonathan McKinney

**Affiliations:** Departments of Philosophy and Psychology, University of Cincinnati, Cincinnati, OH, United States

**Keywords:** embodied cognition, complementarity, Japanese philosophy, ecological psycholgy, enactivism

## Abstract

The enactive and ecological approaches to embodied cognitive science are on a collision course. While both draw inspiration from similar views in psychology and phenomenology, the two approaches initially held seemingly contradictory views and points of focus. Early enactivists saw value in the ecological approach but insisted that the two schools remain distinct. While ecological psychology challenged the common foes of mental representation and mind-body dualism, it seemingly did so at the cost of the autonomy of the agent. This is evidence that the early enactive and ecological approaches told different stories about how agents and environments interact. Whereas the enactive approach broadly focuses on agency and the organism’s resilience to environmental perturbations, the ecological approach insists that organisms are best understood in terms of the organism–environment system and at the ecological scale. Historically, this tension created space for harsh criticisms from both sides and for some ecological psychologists to dismiss enactivism altogether. Despite their differences, both approaches use dynamic systems theory to explain the interactions between embodied agents and the environment or contextual milieu in which they are embedded. This has led some scholars to focus on the complementary elements of each approach and argue that the two schools are allies, thus rejecting the historical disagreements between the two approaches and calling for an ecological–enactive synthesis. The attempts to synthesize the approaches are noteworthy and should be considered steps in the right direction but are potentially problematic. If the two schools are merely synthesized to some form of ecological–enactivism, then something of value from both approaches could be lost. This is analogous to the hasty comparison between two seemingly similar schools of thought found in early attempts at East-West comparative philosophy. I argue that the relationship between the enactive and ecological approaches is both complementary and contrary and is thus best understood in terms of complementarity. Given the complexity of complementarity I will unpack the notion in steps. I will begin with the exploration of analogous concepts in Japanese Philosophy and gradually build a lens through which both agent environment and ecological enactive complementarities can be understood.

## Introduction

The alliance between the enactive and ecological schools is well established, but their differences are not well understood. Broadly speaking, Enactive Cognitive Science focuses on autonomy and the rejection of mind-body dualism (Varela et al., 1991/2016; Thompson and Stapleton, 2009; Thompson, 2010; Di Paolo, 2018) and Ecological Psychology focuses on the richness of perceptual experience and the rejection of mind-body and body-world dualisms (Gibson, 1966, 1979; Richardson et al., 2008). Both schools of thought claim that the embodied mind is embedded in its environment, although their stories are not quite the same (see Ward and Stapleton, 2012). I propose that the ways each school of thought relates the mind to the world is a crucially informative point of tension between the two programs. Whereas the traditional ecological approach provides a strong account of the organism–environment system, it struggles to explain subjective differences in our embodied experiences. The ecological approach lacks a convincing story about how individuals resonate with some affordances and not others. On the other hand, the traditional enactive approach provides a strong account of human agency and subjectivity, but provides little explanation of how environments constrain and enable action. Early enactivism thus lacks a robust account of an interactive information-rich world. Each only accounts for either the active agent or its interactive world, but not both.

Despite their differences, contemporary enactivism and ecological psychology are converging. Baggs and Chemero (2018) argue that the threads of enactivism that follow Merleau-Ponty should be synthesized with the ecological approach to form *Radical Embodied Cognitive Science* through the use of non-linear dynamical systems theory (see also Chemero, 2009). On the side of enactivism, Stapleton (2016) and Di Paolo (2016) argue that the two schools are allies, and are separated only by the *uncanny valley* of misunderstanding (see also Thompson and Stapleton, 2009). I will focus on the cooperative forms of each approach. The strongest case for an enactive–ecological synthesis was proposed by Costantini and Stapleton (2015) and Di Paolo et al. (2017) when they embed the enactive agent in the ecological organism–environment system by reframing enactive constructivism in terms of honing in on relevant affordances in an overabundant environment. While an ecological–enactive synthesis is promising and should be explored, it fails to adequately accommodate the theoretical and historical differences between the two approaches.

Instead of synthesizing the two, the enactive and ecological approaches should be analyzed with the tools of comparative philosophy, where each side is preserved as they come together to create something new. Building upon the works of Stapleton (2016), Di Paolo et al. (2017), and Baggs and Chemero (2018) I argue that the relationship between the enactive agent and the ecological organism–environment system is one of complementarity, where both sides are mutually co-dependent yet persist as individuals in tension. Understanding the ecological–enactive and agent-world relations in this way preserves the subjectivity of enactive agency, the objectivity of the ecological agent–environment relation, the tensions entailed by the relation, and mutuality between them. Unpacking the enactive∼ecological complementarity will require tools from various traditions and is best understood when broken into steps.

The myriad dynamics at play between an agent and its world extends well beyond the scope of this paper. As a result, I will limit my analysis to the interaction between the agent and the world in the present moment, as this will frame the complementarity of the two approaches in a way that’s accessible to the tools of Japanese Philosophy. This relates directly to the enactive timescales of experience introduced by Varela (1999) and developed by Gallagher (2017, pp. 8–9), where the present moment is analyzed at the *integrative scale.*

**FIGURE 1:**
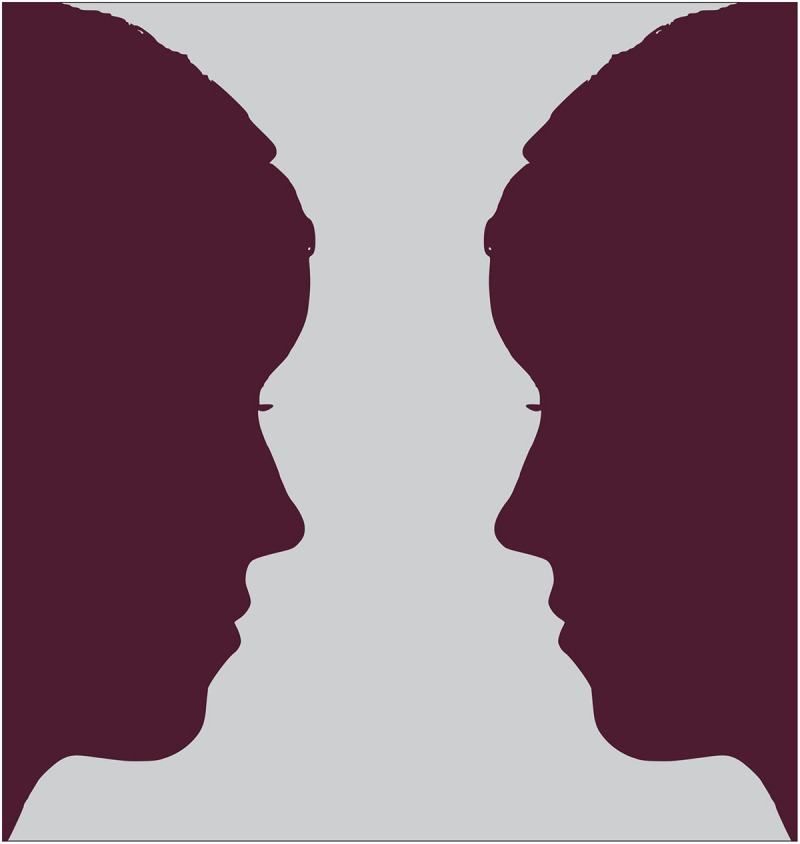
This is an example of the figure–ground relation portrayed by the psychologist Edgar Rubin (See Pind, 2014, pp. 214-218). Interestingly, Rubin was influenced by Niels Bohr, the physicist responsible for complementarity in quantum mechanics. In likeness of Rubin’s Vase and other figure–ground images, Niels Bohr chose the Chinese yin-yang symbol for his coat of arms upon being recognized for his achievements in Denmark. It read “Contraria sunt complementa (opposites are complementary).” For more information about the crossover of figure–ground in perception, complementarity in epistemology, and nonduality in non-Western philosophy, see Pind (2014, pp. 204-210). Image attribution – Nevit Dilmen/CC BY-SA (https://creativecommons.org/licenses/by-sa/3.0).

Kelso and Engstrøm (2006) have argued that complementarity (signified by the symbol “∼”) is difficult to grasp, yet present in our everyday experiences of the world. I have argued elsewhere that the Japanese philosopher Nishida Kitarō’s work is uniquely placed to help mediate between the enactive and ecological approaches and shed light on complementarity (McKinney et al., in press; see also Yusa, 2002, pp. 185–187) (we explore how enactivists, ecological psychologists, and Nishida discuss habits). This argument rests upon the existing works comparing William James and Merleau-Ponty and Nishida (see Yuasa, 1987; Maraldo, 2017; Loughnane, 2019). Nishida’s radical nondualism is built upon the continuity of discontinuity and thus resembles complementarity, and can be used to frame the ecological and enactive approaches through the structure of the present moment. I will apply Nishida’s dialectical analysis to explore the relationship between the enactive agent and the ecological environment as a form of mutual negation. From this comparison, I conclude that the enactive agent and the ecological world can be understood in figure–ground (Figure 1) terms. While this abstraction does not exhaust the relationship between the two approaches, it sheds light on the complementary and contrary nature of the two approaches and invites further consideration of Japanese philosophy in cognitive science.

**FIGURE 2:**
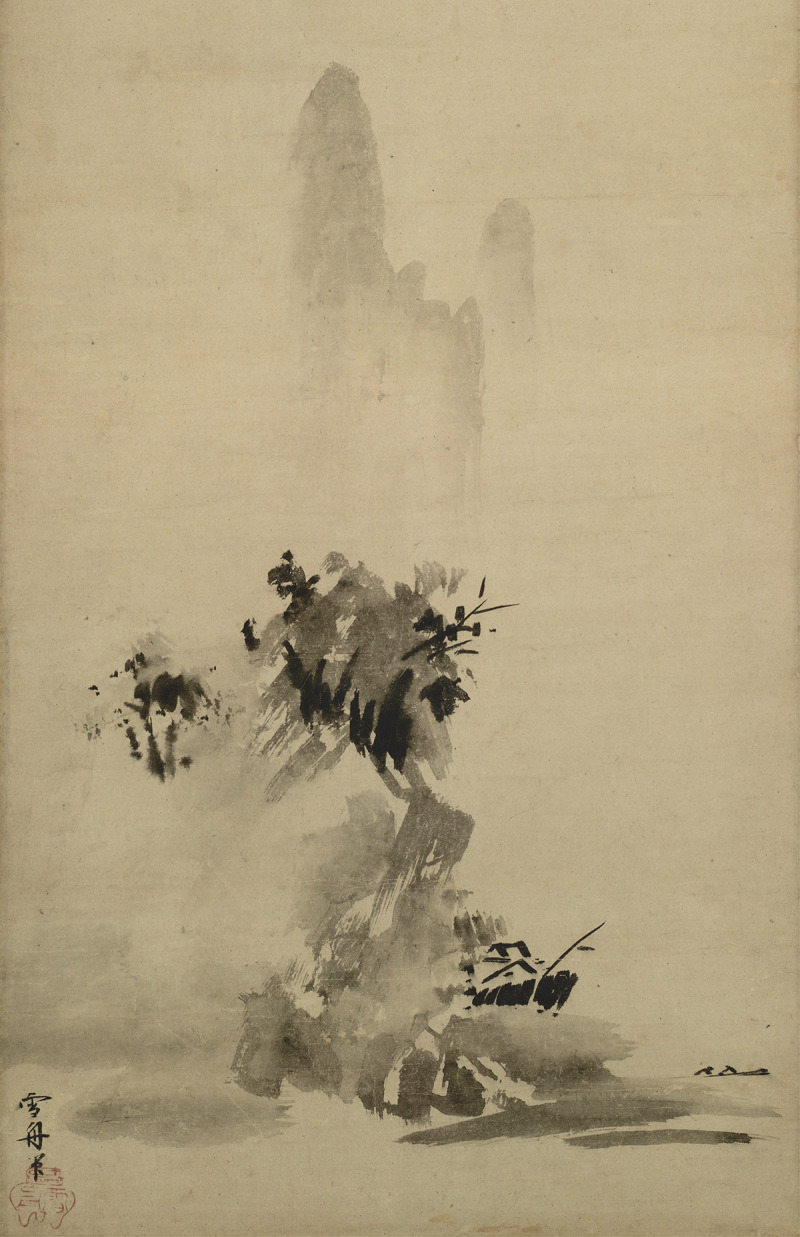
Splashed Ink Landscape (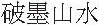) Haboku sansui by Sesshū Tōyō (1420-1506). Image attribution: Sesshū (1495), Tokyo National Museum, Tokyo, Japan (Wikimedia Commons).

I will then consider the insights of the Zen Master Dōgen, who developed a notion of nonduality to navigate perplexing contraries, to develop the figure–ground relationship from the mere duality of figure and ground to the complementary contrary, *figure∼ground.* This is built upon Dōgen’s tripartite elucidation of affirmation, negation, affirmation. To exemplify this, I briefly compare the figure–ground abstraction in Figure 1 with the figure∼ground complementarity in Figure 2. Whereas complementarity and nonduality can be seen in the idealization of Figures 1, 2 is a representation with more direct relation to the real world. I conclude by applying Dōgen’s wisdom to the complementarity of *ecological∼enactivism* and by making suggestions for future research into cross-cultural cognitive science.

## The Enactivism–Ecological Alliance

Whereas enactivists focus on the lived body of agents, ecological psychologists focus on the world agents inhabit. Ecological psychologists frame the agent–environment relationship in terms of the opportunities for action that are available for the agent. This is not to say that ecological psychologists do not care about the agent, but that they do not ground their explanations in the head or body of the agent. Enactivists, on the other hand, prioritize the experience and poesis of the *operationally closed* agent as she enacts her world (Di Paolo et al., 2017, pp. 111–120). The enactive agent is autonomous, meaning that it is resilient to the push and pull of environmental forces, and capable of shaping her world. Agents can create opportunities for themselves to act above and beyond what is available in the environment alone (Thompson and Stapleton, 2009, see also Stapleton, 2016). While the two approaches agree that we are embodied beings, they disagree about the relationship between the agent and the world (Isaac and Ward, 2019).

Varela et al. (1991/2016) briefly considered the similarities between enactivism and Gibson’s ecological approach and concluded that the two are distinct. They recognized the potential for ecological psychology to challenge mind-body dualism, and thus representational cognitive science, but considered the ecological approach to be too focused on the environment. For early enactivists, the enactive agent constructed her world through her coupling with it at different timescales, but the ecological world was pre-given and thus not the product of an agent-constructed process. This kind of criticism of early ecological psychology is commonplace. Chatterjee (2011, pp. 254–257) and Ishida (2015, pp. 136–137) argue that Gibson’s approach is too focused on perception and information pick-up, and thus fails to capture the complexity of agent-world interactions. Baggs and Chemero (2018) argue that the origin of the disagreement between the enactive and ecological approaches results from opposite ends, where enactivists began with the epistemology of embodiment, ecological theorists began with the ontology of embodiment. They insist that,

Both types of explanation are necessary: the ontological strategy explains how structure in the environment constrains how the world can appear to an individual, while the epistemic strategy explains how the world can appear differently to different members of the same species, relative to their skills, abilities, and histories.

This is reflected in the philosophical commitments entailed by each approach. Whereas ecological psychology adhered to a kind of Jamesean monism, the enactive approach aimed to carve a path between dualism and monism. While this distinction held for early versions of the ecological and enactive approaches, it has become less relevant as they developed further. Whereas early enactivists rejected monism, contemporary enactivists have adopted neutral monism (Thompson, 2001, see also Chemero, 2009, pp. 184–186). This is significant because contemporary ecological psychologists also accept neutral monism, and argue that this is the result of the convergence of enactivism and ecological psychology through the use of dynamical systems theory (Silberstein and Chemero, 2015, see also Silberstein and Chemero, 2011).

While there are some skeptics who reiterate the historical differences between the two schools, it seems clear that the two research programs need each other. Stapleton (2016) argues that enactive principles are compatible with the ecological approach and that their historical differences are overemphasized. Di Paolo et al. (2017), citing Thompson and Stapleton (2009), paint the best contemporary picture of the relationship between enactive epistemology and ecological ontology. They argue that Gibson’s theory of affordances, and Chemero’s (2009) development of it for *Radical Embodied Cognitive Science*, overcomes mind-world dualism. Like Baggs and Chemero (2018), Di Paolo et al. (2017) argue that the tension between the two approaches arises for ecological psychologists from the lack of an appreciation of how individual agents perceive the same basic ecological scene differently. What each person sees available to them (i.e., grasps through direct perception) is different based on the context, and what abilities the agent has cultivated.

Importantly, this phenomenon is well established in the ecological approach by the canonical works of Proffitt et al. (1995) and Proffitt et al. (2003). They demonstrate that an agent’s perception of a hill’s climb-ability varies with encumbrance. A hill to be climbed appears steeper to agents carrying more weight. Gallagher (2017, pp. 155–156) argues that this evidence can be used by enactivists to help explain the relationship between social burdens and the approachability of social situations, or social affordances. A chair may afford sitibility in general, but there are many contexts in which agents will not see a particular chair or set of chairs as sitible. While it is significantly more difficult to vary social pressure than physical encumbrance in a lab, human agents are social and live in complex contextual social worlds. Whereas some ecological psychologists would opt to avoid such complications, enactivists recognize that social concerns like feeling unwelcome are ubiquitous and must be at the forefront of any science of the embodied mind.

### Enactive Agent, Ecological World

While this rift persists between the two approaches, Di Paolo et al. (2017) propose the most radical and comprehensive attempt at integration. They begin with the claim that the ecological and enactive schools seem to simply look past one another. The enactive focus on autonomy and operational closure leads ecological psychologists to believe that the enactive agent is being characterized as the source of its world. Di Paolo et al. (2017) solve this problem by embedding the enactive agent in the world of ecological affordances. They claim that the ecological rejection of the impoverishment of the stimulus, and thus one major call for mental representations, should be accepted by enactivists. Indeed, they claim that such a world is overflowing with information.

We agree with ecological psychologists when they highlight that real environments are rich enough to access directly their relevant meaningful aspects. We think they are in fact too rich, in that sense-making always involves a massive reduction of all the environmental energies that might affect the agent, to those within the dimensions of biological, sensorimotor, and social historically contingent meaning (p. 227).

They diagnose the disagreement between the two schools on the ecological side as stemming from the failure of ecological psychologists to change how they treat the information-rich world. According to Di Paolo et al. (2017), the world of affordances should be understood to extend to the social dimensions of experience.

Ecological experiments tend to de-emphasize subjectivity, and thus fail to develop the notion of affordances to include social and cultural context. Recent work in ecological psychology by Nalepka et al. (2015) has begun addressing these issues by exploring intersubjectivity in terms of coordination dynamics and interpersonal synergies (Chemero, 2016). While it is valuable to control messy variables in laboratory conditions, one must acknowledge the costs of doing so. The world is messy and social and economic hierarchies permeate our lives, shaping our experiences of the world and the opportunities available to us. In Di Paolo et al. (2017)’s interpretation, the landscape of affordances available to an agent is not contingent upon the agent itself, but is modulated by the agent’s history, experiences, and social context.

In this light, the enactive agent is not creating her world from her mind or body, but carving her world of experience from the overwhelmingly informative world. The distinction is subtle but shows how two different views of embodiment have converged. It’s helpful to consider this subtle distinction through an example. Consider a scene in a typical coffee house. There is a coffee machine, staff operating the machine, chairs, tables, and cups of coffee. If one were relatively familiar with scenes like this, it would be an easy environment to navigate, find a seat, and achieve the goal of acquiring and drinking a cup of coffee. If we were to consider the scene in ecological terms, the surfaces of the environment would be relatively accessible to us through what they afford us. Chairs facilitate sitting and cups afford sipping for agents that move through the visual scene. Agent’s movements reveal invariant structures and opportunities available to them.

If we consider the same scene through Di Paolo et al. (2017)’s view, it becomes somewhat more complicated. Whereas all empty seats afford sitibility in general, which chairs appear sitible depends on the context. Whereas some seats are open to us, others are not. One easy example of this is the *sippability* of another person’s cup of coffee. Any two identical coffee cups, filled with the same amount of coffee, should be equally sippable, yet we do not see other people’s coffee as sippable in most cases. This is because of the complex contextuality of the human world. While it is complex to model and reproduce, it is an everyday experience for regular coffee shop goers.

Zooming out from the two cups of coffee, there are places in the coffee shop that a customer is not welcome even though they have surfaces and tools that humans are capable of engaging with. The cups behind the coffee bar are within our power to reach, yet in most situations we will wait for someone else to get them for us, or ask for temporary permission in rare cases. Interestingly, in many cases, we perceive the approachability of the staff as a means of accessing the cup, rather than the path to get the cup ourselves. In this and many other cases, the social path is more readily available than the sensorimotor path. This has crucially important implications for how we understand the ecological landscape and where the enactive agent comes into the picture.

The first implication is that there can be spaces which do not seem readily accessible to people, even though the visual scene devoid of social context is accessible. Much like how the employee-customer distinction informs the social affordance landscape of coffee shops, other forms of discrimination fundamentally shape what affordances we have access to. Young (1980) argues that there are places women won’t go and activities that women avoid because of societal pressures and patriarchal oppression. While these are not the same kind of contextual barriers, understanding social affordances in this way sheds light onto what can be done to make space for everyone. Enactivism and ecological psychology can meaningfully contribute to our understanding of embodiment, and all of its positive and negative entailments.

The second implication is that we can create space for others and shape our social-contextual worlds. Being able to create space to act is the strongest contribution that the enactivists can bring to the ecological approach. If we step back into the coffee shop example, there’s an important distinction between actions in the present moment and actions that take place over longer timescales. While it is true that I do not see the space behind the bar as walkable as a customer, I can see possibilities to cultivate the social space for myself through my friendships with the staff or my plans to apply for a job at the coffee shop. I could join the coffee community in the area and become familiar with the tools, norms, and processes of that group. In doing so, I may be welcomed behind the coffee bar, and thus break the barrier that I faced when I was merely a customer passing through. Likewise, it is possible to change the social environment of a space to be more inclusive. Ultimately, the social world is messy, but this interpretation opens the door for exploring the importance of the environment for our experiences of welcomeness. Enactivism already explores the perception of and action at multiple timescales, and an enactive–ecological perspective could do so with a strong grasp of the ontology of places and our relations to them.

In the first sections of this paper, I have sketched some points of contact and contention between the enactive and ecological approaches, as well as traced their converging paths. While the two schools often look past one another, I have provided further support for their allegiance and continued cooperation. Next, I would like to provide a lens for understanding complementarity as it plays out in both ecological∼enactivism and agent∼world relations. To do this, I will focus on the moment of present experience, as it serves as a paradigmatic example of an agent *enacting her world* while embedded in the ecological organism–environment system.

## Nishida and the Frame of the Present

Like Gibson, Nishida aimed to subvert subject-object and epistemology–ontology dualisms. To do this, Nishida structured the present moment of experience in both spatial and temporal terms as the continuity of discontinuity. For Nishida, the agent is embodied and embedded in its world, yet an individual.

In order to best understand the paradoxical nature of lived experience, Nishida brings together traditional philosophical dichotomies like spatiality vs. temporality, mechanistic materialism vs. teleology, and subjectivity vs. objectivity. Each dualism meets in and forms the structure of the present moment of embodied experience. For Nishida, the past and the future are both active in the moment of experience. The embodied agent is undoubtedly the result of her past. As an embodied being, she has grown up embedded in a social and cultural context which have shaped her experiences. Likewise, her biological history involves things like the evolution of her species and the constraints and abilities of her body and bodies like hers. Nishida’s agent is not merely the product of her past; she is formed by her past into a being capable of forming herself, her world, and others like her. For this to be possible, Nishida (1958) argues that the agent’s past must confront her environment and present circumstances, which serve as possibilities to enact her future.

[I]n the historical-social world subject and environment confront each other and form each other. This means that past and future oppose each other in the present, as unity of opposites, and move from the formed toward the forming (p. 184).

Crucially, Nishida characterizes the dialectical tensions of experience as a process of mutual negation. This is strikingly similar to the claim made by Di Paolo et al. (2017, p. 227) that sense-making entails reducing the abundance of information available in the ecological world into an agent-relevant and actionable scene. Whereas enactivists argue that agents must overcome environmental forces to express themselves, Nishida de-emphasizes the agent and argues that poesis is impossible without a place (*basho*) in which to act. Importantly, the basho of our embodied actions is not merely the ground we stand on, but more akin to an event in which we are embedded. Kasulis (2018, pp. 466-467) refers to *basho* as a *how* rather than a what, because it is how the dynamic processes of life come about. It helps us understand how agency like ours is possible ontologically.

In Nishida’s view, self-expression is achieved through the negation of the environment, but the environment is an irreducible part of that self-expression because the two cannot be meaningfully separated. This is important because it could spell out a way to pull the enactive–ecological dialectic in the direction of ecological principles through a focus on the ontological side of the ontology–epistemology dyad. This kind of constructive opposition is an important aspect of the complementarity between the enactive and ecological approaches.

I propose that the best way to understand the relationship between ecological psychology and enactivism is to bring together their conceptions of embodied perception and action in the present moment of experience. In the moment of experience, both the past (the historical agent and the historical world) and the possible future (affordances or the environmental invitations to act) play a role. The history of the organism and the world, here analogized with the enactive conception of the evolutionary development of the *autonomous sense-making* agent, and the available space and opportunities for action, here analogized with the ecological theory of affordances, meet at the center of Nishida’s present moment of experience. Both play fundamental roles in the agent’s *poesis.*

### Nishida and Artistic Expression

According to Nishida, the expressive act is not an enaction from the agent upon the environment, but the result of both the agent and the circumstances in which she is embedded (see Section 4.1 of Maraldo, 2015). While it is accurate to credit the painter with her creation, Nishida would argue that the painting should also be understood as an achievement of the world itself. For Nishida, creativity and consciousness are essentially embodied, but require the continuity of discontinuity of the agent and the world. In any given moment, the agent is the embodiment of her biological-social history and a figure upon the ground of her environment. At the same time, the agent would be unable to make meaningful choices and express herself if she were not faced with the teleological pull of the future. The same moment can be understood both by taking the agent as the figure and the world as the ground, and the world as the figure with the agent as the ground.

In his later works, Nishida develops agent-world nondualism ontologically. For Nishida, the place (*basho*) in which agents are embedded are not merely the grounds she stands upon (See Heisig et al., 2011, pp. 649–661). Embeddedness entails the distribution of cognition where the agent and the event she is enacting cannot be meaningfully distinguished. As a result, it would be a mistake to emphasize the agent’s creativity without recognizing the constitutive effects of the myriad events and processes at work which make it possible for artists to be [Kasulis (2018) clarifies Nishida’s notion of *basho* as a field akin to gravitational or electromagnetic fields that is best understood as a *how* rather than a *what*].

If we consider the relationship between the artist and her environment in this way, it forms a kind of figure–ground relation where the artist’s self-expression and the aesthetic scene mutually depend on each other. While this is an abstract picture, it demonstrates the mutual importance of both sides of the agent-world relation. This is exemplified in Nishida’s early works when he explores the fusion of the subject and object through the paintings of Sesshū Tōyō, see Figure 2.

At that point we can say that things move the self or that the self moves things, that Sesshu painted nature or that nature painted itself through Sesshu. There is no fundamental distinction between things and the self, for just as the objective world is a reflection of the self, so is the self a reflection of the objective world [This passage is from Nishida (1990), p. 135, and quoted from Loughnane (2019), p. 153.]

While the seeds of Nishida’s nondualism can be found in his early work *Inquiry into the Good*, he shifted his focus from the epistemological unity of the artist and the world to the ontological nonduality of place (*basho*) in his later works (Kopf, 2010, pp. 144-151; See also Nishida, 1987). This is crucially important for understanding complementarity and for unpacking the peculiar relationship between the ecological and enactive approaches. As I have argued elsewhere, by resisting the focus on the subjectivity characteristic of phenomenological agents, Nishida’s approach pushes our analysis in the direction of ontology, and thus toward the ecological approach (McKinney et al., in press) (Nishida’s work is a useful lens through which the enactive–ecological relationship can be made clearer). This is important because the best attempts to integrate enactivism and ecological psychology have come through the adaptation of the ecological approach to accommodate for the autonomy of the enactive agent, without a similar accommodation for the inseparability of agent–environment systems. While Di Paolo et al. (2017) accept and enhance the structure of the ecological world, they do so without a clear account of the distribution of cognition, which is a fundamental tool for dismantling the poverty of the stimulus position and weak forms of the extended mind hypothesis (Steffensen, 2011, see also Cowley, 2011, Cowley, 2014). While it may seem counterintuitive to accept that the world is the co-author of a poem, failing to acknowledge the real impact one’s place in the world has on one’s art, or even one’s access to artistic expression, is problematic. To unpack this further, consider the relationship between the agent and the world in terms of the figure–ground abstraction in Figure 1.

## Enactive Agent-Ecological World as Figure–Ground

In order to best understand complementarity, we should proceed in steps. The first aspect of complementarity worth exploring is the perplexing way two things can co-exist as a complementary yet contrary pair. To that end, it is helpful to visualize the relationship between the agent and her world in terms of figure and ground for several reasons. If one focuses on the figure, the ground fades from view. Likewise, if we focus on the background, we lose focus of the figure. In the figure–ground image in Figure 1, the image is neither merely of a vase nor of two silhouetted faces. It’s tempting to say that it is simply an image of both, but this fails to recognize the ambiguity of the image itself. To capture the figure–ground image, and thus to distinguish it from images of a pair of unambiguous images, we must recognize that the images are co-dependent. To have one necessitates the other. Considering the images in Figure 1, both the vase and the pair of faces can be the figure and the ground, which forms an absolute mutuality between the two. For our comparison, this accomplishes two things. The first is that figure–ground images represent how two images can depend on each other to exist. The second is that figure–ground relations represent an abstract equality in that neither side of the relation overtakes the other. The Rubin’s Vase image in Figure 1 is useful for representing two equally recognizable idealized images in tension, but is ultimately too abstract to capture non-idealized complementarity relations. In order to understand the mutuality and tension between an artist and her work of art or an agent and her world, we must develop the figure–ground relation further in the next step.

Although the figure–ground relation is a useful abstraction, problems remain. In order to further clarify complementarity, and hopefully bridge the uncanny divide between enactivism and ecological psychology, the figure–ground relationship should be considered in less abstract terms. Consider figure–ground relations in visual scenes in the real world. In cases when an object is in focus and well framed by its surroundings, the two are integral yet not equal contributors to the scene. In fact, the dynamic tension between the two is an essential and informative aspect of what being in relation to one another means. This is exemplified by Zen paintings.

In Zen paintings, objects of importance must overcome space to emerge as a figure, yet the image is not dominated by the figure alone. Much of the canvas is left blank on purpose, and images of prominent figures like mountains are obscured by clouds or fog. The empty space filling the canvas makes it possible for us to comprehend the partial emptiness of the fog obscuring the figure. Unlike the figure–ground abstraction (Figure 1), Sesshū Tōyō represents the dynamic tension between a multi-dimensional figure and the ground of the contextual scene and emptiness in several famous landscape paintings; one can be seen in Figure 2. Whereas the vase-face duality was an important step for contextualizing mutuality, the mountain∼fog nonduality is more useful. Whereas Figure 1 is a two-dimensional image of a vase silhouette that forms two identical faces, Figure 2 is a painting of a multidimensional mountain in a foggy scene. The latter emphasizes the importance of both spatiality and temporality for our lived experience of the world.

### Zen Philosophy and the Teachings of Nonduality

Having analogized the enactive–ecological relationship, and thus the relationship between the agent and the world, in figure–ground terms, we are now in position to fully realize the complementarity of their allegiance. This involves stepping beyond the abstract two-dimensional figure–ground image and attempting to apply this analogy in real life. This last step is designed to respond to the thoughtful criticisms of empirically minded enactivists and ecological psychologists who may appreciate the figure–ground analogy, but don’t see how to use it. To help escape the abstract in favor of the real, we should turn to the insights of the practical teachings of the Zen Master Dōgen.

In Zen, apparent contradictions and dichotomies are opportunities to teach others and learn about the world. This is exemplified by Dōgen in his writings about *nonduality* [It is important to note that Evan Thompson (2020) has rightly cautioned against hastily comparing Buddhist nonduality and the rejection of Cartesian mind-body dualism. Buddhism has a long and rich history that should not be hastily sampled out of context. I propose that Dōgen’s teachings of nonduality can be deployed for their pedagogical insights without committing cognitive scientists to something akin to a Zen metaphysics]. In the introduction to the text *Moon in a Dewdrop: Writings of* Zen master Dōgen (1985), Kazuaki Tanahashi introduces the following passage to exemplify what he calls Dōgen’s *Anatomy of Nonduality.*

An ancient buddha said, “mountains are mountains, waters are waters.” These words do not mean that mountains are mountains; they mean mountains are mountains.” (see also Schroeder, 2010, pp. 133–142)

While Dōgen’s purpose is soteriological, the structure of his reasoning is most important for this project. Tanahashi writes, “Dōgen’s demonstration starts with an affirmative statement, then negates the affirmation, and concludes with a negation of the negation, which is a positive statement.” For Dōgen, nonduality involves the coming together of contradictory perspectives into a realization of the world *as it is*, which is the unity of the contraries. Tanahashi compares Buddhist reasoning with Western dialectics, wherein one progresses from a contrary pair to a higher synthesis. For Dōgen, each step of the reasoning process entails every other step, and there is no progression from lower to higher reasoning. Consider Dōgen’s mountain. If one were to approach the mountain as an object, its emptiness is part of the experience. Likewise, if one approaches the mountain as a scene of interdependent relations, the stability of the mountain as an object is part of the experience. The mountain exists as an object and exists as an empty process, even though emptiness and substance are contradictory modes of being.

Dōgen is demonstrating the paradoxical nature of reality found in the mundane experience. He urges his students to realize that the dichotomy between existence- and emptiness-focused perspectives yield the same conclusion: the mountain is both substantive and empty. The pedagogical takeaway from Dōgen is that no matter which perspective you begin with, the affirmative, the negative, and the continuity of discontinuity are all entailed. If you focus on the importance of the ground for the existence and prominence of the figure, you will realize the co-constitutive nonduality of the figure and the ground. Likewise, if you focus on the importance of the figure for the shape and existence of the ground, you will be faced with the figure–ground nonduality.

Having considered the two-dimensional representation of the figure–ground relationship and Dōgen’s notion of nonduality, we should now reconsider the figure–ground relationship in Figure 2. Sesshū Tōyō’s famous painting emphasizes the relationship between emptiness and form. The figure, here a mountain, is painted intentionally as partially obscured by mist or fog. This exemplifies the tension between the figure and the ground and emphasizes the Zen notion of nonduality. It does this through both temporal and spatial dimensions, as the empty space around the figure draws the attention of the viewer to it, yet it partially obscures the figure as if it could disappear into the fog. The fog and blank space are designed to subvert our grasp of the importance and permanence of the mountain as the figure, without reifying the importance and permanence of emptiness as the figure. The two are co-dependent upon each other for their being, yet they are not equal. This inverts and challenges our undue focus on the figure and disrupts the sense that the figure and ground should be considered as abstract equals. In contrast with the abstraction of Figure 1, the tension between the mountain and the emptiness resembles the tensions of the real world of our lived experiences. Zen teachings are often aimed at shifting the practitioner’s mind away from abstractions and back into the world in all of its complexity.

The two-dimensional figure–ground image is useful for representing the complexity of mutuality that persists through tension. This idealization of mutuality fails to account for the complex dynamics at play in concrete constitutive relations, like those found between an agent and the environment she is embedded within. The complex relationship between them is dynamic, meaningful, and ever changing. For our purposes, the figure–ground abstraction is comparable with the initial attempts to synthesize ecological psychology and enactivism seen in the works of Stapleton (2016), Di Paolo et al. (2017), and Baggs and Chemero (2018). The enactive–ecological synthesis obscures the historical differences between the two approaches. This is significant because it impacts the kinds of questions that researchers ask and the experimental methods they use. It is useful to maintain some tension between the enactive and ecological approaches because there are times it is best to focus on the subjectivity of the agent, thus neglecting the objective universalizability of the empirical laboratory. Likewise, there are times it is best to neglect subjective differences and constrain autonomy to uncover the invariant structures of the ecological environment. This is the best way to frame the relationship between the ecological world and the enactive agent, and ultimately the complementarity of the two approaches. The complementary contrary ecological∼enactivism is more explanatory than either approach taken in isolation or a partial synthesis of the two.

Dōgen’s teachings of nonduality emphasize the importance of being able to approach contraries from multiple perspectives while still arriving in the same place. As a Zen teacher, Dōgen was pragmatic and could deploy and defend either perspective of the contrary dyad. This dispels the abstraction of symmetry found in figure–ground abstractions and instead makes room for complementary relations with the capacity for opposition and asymmetry. Life and the world of human experiences are messy, complex, and rarely well balanced. As a result, it is crucial that our models of living are equally complex and able to exhaust the many counterintuitive interactions life entails. One important takeaway from Dōgen’s teachings is that it may be possible, and in many cases it may be essential, to understand the ecological∼enactive complementarity with more emphasis on one perspective over the other.

In cases involving subjectivity and autonomy, an enactive framework is likely most explanatory. In cases involving environmental invariants and embodied synergies, an ecological framework is likely more useful. These are arguments already being made by ecological psychologists and enactivists to challenge one another. What remains is to realize that both sides entail the other, especially when considering the converging works of Stapleton, Baggs, Di Paolo, Chemero, Thompson, and their collaborators. The last step is to embrace the fact that the complementary similarities and contrary disagreements between enactivism and ecological psychology entail a complementarity relation, and thus calls for the shift from ecological–enactivism to ecological∼enactivism.

## From Ecological–Enactivism to Ecological∼Enactivism

The enactive and ecological approaches are allies, but their past disagreements and the prospects of future collaboration are complicated. The works of Stapleton, Baggs, Di Paolo, and Chemero are converging, yet others are reluctant to follow. At least one reason for this is the counterintuitive nature of complementarity. The notion of nonduality in Japanese Philosophy provides an informative framework to engage with complementary contraries like epistemology–ontology, self-other, and self-world. This is particularly helpful for contextualizing the relationship between the enactive agent and the ecological organism–environment system. I first proposed that the agent-world relationship, and thus the enactive–ecological synthesis, should be understood in figure–ground terms. This helped contextualize the importance of the agent’s embeddedness in the environment, and the non-decomposability of agent-environment systems. While the figure–ground relationship is useful, it’s too abstract. To move away from models and abstractions and toward the world of everyday experience, I next invoked Dōgen’s tripartite method of reasoning to develop the figure–ground relation into the nonduality or complementarity: figure∼ground.

The figure∼ground relation is one of complexity, interdependence, and double-negation and can be used to help frame the agent∼world relation entailed by ecological∼enactivism. This frame provides two key takeaways. The first is that the complex dynamics of living as an enactive agent embedded as a part of the ecological organism-environment system entails an ongoing tension between the autonomy of agency and the obstacles and opportunities of an information-rich world. While this relationship can be complementary and symmetrical, it can also be asymmetrical, where one side temporarily overtakes the other. The second is that the tension entailed by the agent∼world complementarity necessitates a similar tension between the enactive and ecological approaches, and thus a shift from an ecological–enactive synthesis to an ecological∼enactive complementarity.

## Author Contributions

The author confirms being the sole contributor of this work and has approved it for publication.

## Conflict of Interest

The author declares that the research was conducted in the absence of any commercial or financial relationships that could be construed as a potential conflict of interest.
